# Evaluating cognitive, metacognitive and social listening comprehension teaching strategies in Kuwaiti classrooms

**DOI:** 10.1016/j.heliyon.2019.e01264

**Published:** 2019-02-27

**Authors:** Asma F.T. Al-Azzemy, Dina A.H. Al-Jamal

**Affiliations:** Department of Instruction & Curricula, Yarmouk University, P.O. Box 566, 21163, Irbid, Jordan

**Keywords:** Education

## Abstract

Listening comprehension strategies play an important role in the development of related language skills and process of language acquisition. These self-regulated strategies are focused to enhance learning comprehension and their consequences. The study aimed to evaluate the listening comprehension strategies covered by Kuwaiti ninth grade target English textbook with adherence to the teaching practices of EFL teachers. Qualitative research approach has been adopted with content analysis to assess the listening comprehension strategies. 38 teachers were selected from 16 Kuwaiti schools and involved a textbook of ninth grade. High practices have been achieved from all listening comprehension strategies. Inferencing and practicing sounds revealed as the viable strategies covered by Kuwaiti textbooks. Highest mean score has been accomplished by cognitive strategies (M = 1.87, SD = 0.16); whereas, low mean scores were examined for metacognitive strategies (M = 1.74, SD = 0.29). Evaluation strategy was considered as the most frequent application examined in all teaching units. The listening comprehension abilities are significantly affected from cognitive and metacognitive strategies to provide guidelines for EFL (English as a Foreign Language Teachers) to improvise listening comprehension skills of students. The study has concluded with the need of incorporating explicit strategy training in national listening curriculum in Kuwaiti schools to endow teachers with some guidelines to enhance learners' listening comprehension.

## 1 Introduction

Learning comprehension strategies hold a vital position in second language attainment research. Language learning theories are devised on the basis of cognition theories to approach diverse perspectives of classroom practice. Thereby, this ability is regarded as the core step towards language communication (Serri et al., 2012). The ability of learners is perceived by understanding, reading passages, and expressing themselves in writing, which relates them to the development of listening abilities. The development of learners allows them to comprehend the essence of aural input and associate it with the contextual information. Individuals spend around 30–40% of their time communicating with each other through aural approach. The listening activities contribute a fundamental part in developing language learning and communication. Therefore, the time invested in commuting end up in the development conforming to the quality of interaction. The process of listening entails decoding and constructing essence from the verbal and non-verbal messages (Goh and Hu, 2014).

Listening comprehension is the most undervalued skills in second language attainment. These comprehensions have been identified as the active processes; although, learners comprehend it as a passive activity. Listening practices are deemed to help in recognizing the characteristic differences among sounds and comprehending the grammatical structures (Chien and Hsu, 2013). The essence of prosodic proof and language input are significant to apprehend and be able to decipher the communication context. Furthermore, listening is also deemed as a difficult ability to comprehend that may require sufficient mental effort (Tomporowski et al., 2015). Two major steps are entailed in the listening comprehension. The first approach allows listeners to explain the context of the message to other listeners; whereas, the second approach entails receiving, memorizing and repeating the sounds.

The paucity of student's awareness towards understanding the English language has been notified, which restricts the ability of students to achieve their desired objective. Therefore, Kuwaiti students need to be competent towards learning the English language, as the progressive attitude towards the technological development and globalization. Teaching practices in Kuwait can prove to be significant in developing the listening skills of students. Ministry of education has included English language in national curriculum and raised the teaching standards to enhance the abilities of learners to communicate proficiently in English. Moreover, the factors affecting listening comprehensive tend to possess individual differences among the listeners. Therefore, the present study has evaluated the listening comprehension strategies covered by Kuwaiti ninth grade target English textbook with adherence to the teaching practices of EFL teachers. Furthermore, it also analysed the listening strategies and actual practices of teachers.

## 2 Background

Listening comprehension needs involvement of individuals in an assortment of activities ranging between complete comprehension and discrimination of sounds of the speaker's message. Furthermore, assorted procedures are invested by listeners, which endows them to observe and understand language more effectively (Al-Alwan et al., 2013). Listening comprehension is considered as a cognitive ability due to its active and sentient approach (Podhajski, 2016). Listening comprehension strategies differentiate proficient and less proficient language listeners from each other. Escalating strategies assist language listeners in achieving listening activities more efficiently. The positive and imperative influence of listening strategies are effective in listening comprehension of EFL (English as a Foreign Language) learners (Tavakoli and Biria, 2014). Furthermore, the confidence of learners is improved, as well as anxiety is eradicated, through listening to aural input and; therefore, less proficient learners are benefited from the instruction strategies.

According to Rahimi and Abedi (2015), the ability to regulate and monitor the progression, future learning, and performance cannot be achieved without acquiring listening comprehension strategies. Furthermore, language learners who adopt and utilize metacognitive strategies are tend to be more proficient learners. Planning for learning, thinking regarding the learning process, evaluating learning procedures and monitoring production, or understanding are all regarded as listening strategies (Klingner et al., 2015). Here, cognitive, metacognitive and social-affective strategies are the most essential strategies that depend on the cognitive theory (Serri et al., 2012). Among all these factors, metacognitive strategies are considered to be the most effective in developing abilities of learners (Zhang and Seepho, 2013). The impact of development in linguistics and cognitive psychology has encouraged researchers to concentrate on the evidence-based and well-acknowledged approaches to adopt instruction entailing metacognitive familiarity (Rahimi and Katal, 2013). The attributes of strategic learners and the aspects used by learners towards particular language learning tasks have been outlined by scholars (Rasouli et al., 2013; Abdalhamid, 2012).

Listening comprehension abilities are supported by myriad literature embodying learning as effective approach complying to the cognitive necessities, allowing them to further quest for more efficacy (Lysenko and Abrami, 2014). Language learners with high proficiency of metacognitive comprehension are better at storing and processing new information, searching the better approaches to exercise, and reinforcing learning material (Riding and Rayner, 2013). Listening comprehension strategies represent the most fundamental element in establishing abilities of learners in guiding and enhancing the performance. This assertion is being supported by Rasouli et al. (2013), who pointed that cognitive strategies of learners are well-associated with the proficient learning of all learning contexts. Furthermore, the direct and imperative effect of listening strategies on the listening performance is approved in the context of second language attainment, which is known to be determined in particular (Riding and Rayner, 2013).

Successful listening can also be observed on the basis of strategies used by the listener after being taught effective ways of approaching and managing the listening. These activities seek to involve listeners actively in the process of listening. Zhang and Seepho (2013) have focused on metacognitive strategies as planning and consciously implementing appropriate actions to accomplish a specific objective. In addition, listening comprehension strategies have been implemented to regulate the entire learning process. The listening strategies entail the comprehension of language learner to realize the level of implemented strategies and recognize the extent of listening comprehension processes (Zanjani and Izadpanah, 2016). The association among metacognitive instruction and listening performance endows a causal relationship between the learners' effectiveness.

Yeldham and Gruba (2016) have regarded textbooks to be the repository for storing concepts, which supplies opportunities for learners to employ language in assorted contexts. There are three compulsory stages that are observed in the educational system of Kuwait, which includes the primary, intermediate and secondary stage. Among all these phases, primary stage has been deemed to be of interest in the approach. In primary aspect, English is taught as a compulsory along with other seven subjects. English textbooks are adopted on both communicative and structural approaches to meet the required standards of students. Therefore, communicative language as well as vocabulary and grammar are realized to be well-taught in Kuwaiti schools. Textbook is perceived as the most essential requirement for English as a foreign language classroom as it expresses the approach to be detected for learning procedures.

### 2.1 Questions

Following research questions have been devised to answer the specific aims:

**Question 1:** What are the types of cognitive, metacognitive and social/affective strategies occupied by EFL Kuwaiti ninth grade textbook?

**Question 2:** What is the approach of ninth grade teachers to adopt listening comprehension strategies?

**Question 3:** What is the congruence of listening strategies employed by English language teachers in the textbook?

## 3 Materials & methods

### 3.1 Methods

An analytical approach has been employed by using observation checklist and content analysis. The in-depth significance has been provided by utilizing means of qualitative evaluation approaches. Content analysis has assisted in determining the conceptual aspects of the listening comprehension strategies to endow emphasis regarding the listening skills. The population for teacher's observation has been recruited from all the intermediate schools in Hawally Directorate of Education. Subsequently, 16 schools were selected as sample from the total population. The choice of sample was based on random sampling approach. Moreover, 38 teachers were selected from these 16 schools for further evaluation. The ethical approach has been taken from the IRB, Kuwait.

### 3.2 Instrumentation

EFL textbook of ninth grade in Kuwait for the academic year 2015/2016 were considered as the tool of investigation for content analysis. Instruments such as strategy content analysis checklist, reliability, correction criteria, and teacher observation checklist were included to conduct the study successfully. The evaluations were based on clear standards of instructional excellence prioritizing student learning, which helped in determining the reliability of content analysis as well as responses obtained from the respondents. Eight cognitive strategies have been included in the strategy content analysis checklist including; inferencing, elaborating, summarizing, note-taking, highlighting, practicing sounds, and guessing. Furthermore, three metacognitive strategies have been included: planning, monitoring and evaluation. Group work and pair work were included in social strategies to monitor the textbook that encouraged constructive critical feedback and frequent observations.

### 3.3 Study area

Ninth grade has been selected for the assessment as it is the last and senior most grade of intermediate stage in Kuwaiti schools. Therefore, the text book of ninth grade was considered important for the aspects and language learning processes in the study. The listening strategies were observed from the textbook as well as teacher's strategies of language learning process.

### 3.4 Analysis

The checklist for teacher observation strategy was developed on the basis of metacognitive, cognitive, and social strategies. Eighteen items were listed in the final checklist of the teacher observation strategy with a 2-point scale (1 = Available and 2 = Unavailable). The first 9 items in the checklist were related to cognitive strategies; whereas, items 10 to 15 were related to metacognitive strategies. Items 16, 17 and 18 were related to the social strategies. English language teachers were communicated regarding the purpose to establish reliability of the observation checklist. The reliability of the questionnaire was analysed through cronbach alpha with an extent of 0.89. A level of 0.90 was accomplished for reliability of the content analysis. Correction criteria was determined through transcription process where the transcription has been performed on the recordings of listening activities attempted by each student. Furthermore, listening strategies were incorporated to determine the listening skills.

## 4 Results

The types of listening comprehension strategies entailed in ninth grade textbook has been presented in this section. Furthermore, actual and observed teaching practices were also considered in regard to the teachers and to ascribe the delivery of listening comprehension strategies to EFL Kuwaiti learners.

### 4.1 Question 1: What are the types of cognitive, metacognitive and social/affective strategies occupied by EFL Kuwaiti ninth grade textbook?

The results obtained from content analysis addressed frequencies and percentages for cognitive listening strategies in the textbook. It has been examined that Inferencing strategy is the most frequently employed strategy in overall content analysis, as investigated among the teaching units. It has been observed to attribute about 35 frequencies of the total listening texts under study. Increase in frequency and percentage terms have been authorized from the six teaching units. Figuring out evidences, using clues, and illustrating conclusions have been entailed in the inference of cognitive strategies. Furthermore, practicing sound strategy has also been focused from the frequencies and percentages of ninth grade textbook as it comprises the words, phrases, or filling the words in text. Therefore, 30 incidences in all investigated teaching units have been characterized showing five frequencies being captured in each focused unit.

Summarizing and note-taking strategies were moderately associated to the cognitive strategies in the textbook under study illustrating six occurrences in all units. Therefore, textbook activities have been found to be concerned with summarizing strategies focused in each teaching unit. The most significant notions from the text have been entailed in the summarizing activities. The material, making comparison and contrasts, have been outlined in the note-taking activities (Table 1).

**Table 1:** Frequencies and percentages of the infused cognitive strategies in the textbook.

Target English grade 9: student's book			Cognitive strategies															
			Inferencing		Elaboration		Summarizing		Note-taking		Highlighting		Practicing sounds		Guessing		Total	
Module title	No.	Unit title	F.	%	F.	%	F.	%	F.	%	F.	%	F.	%	F.	%	F.	%
(4) Critical thinking	7	Finding answers	10	28.5	3	16.7	1	16.7	1	16.7	2	16.7	5	16.7	1	11.1	23	19.8
	8	Solving problems	5	14.3	3	16.7	1	16.7	1	16.7	2	16.7	5	16.7	2	22.2	19	16.4
(5) Challenges & exploration	9	Emergency & rescue	5	14.3	3	16.7	1	16.7	1	16.7	2	16.7	5	16.7	3	33.3	20	17.2
	10	Journeys	5	14.3	3	16.7	1	16.7	1	16.7	2	16.7	5	16.7	1	11.1	18	15.5
(6) People	11	About our lives	5	14.3	3	16.7	1	16.7	1	16.7	2	16.7	5	16.7	1	11.1	18	15.5
	12	Wishes & regrets	5	14.3	3	16.7	1	16.7	1	16.7	2	16.7	5	16.7	1	11.1	18	15.5
Total			35	100	18	100	6	100	6	100	12	100	30	100	9	100	116	100

Frequencies and percentages are revealed for the metacognitive strategies and illustrated the listening strategies in the textbook (Table 2). Among all these approaches, evaluation strategy has been the most frequent application examined in all teaching units, which characterized 18 frequencies of the total listening texts in the textbook. The strategy has been examined three times in each unit. Wishes and regrets has been examined from the monitoring strategy in unit 12. The modules of planning strategy were characterized in units 7, 8, 11 and 12.

**Table 2:** Frequencies and percentages of the infused metacognitive strategies in the textbook.

Target English grade 9: student's book			Metacognitive strategies							
			Planning		Monitoring		Evaluation		Total	
Module	Unit		F.	%	F.	%	F.	%	F.	%
(4) Critical thinking	7	Finding answers	2	25			3	16.7	5	18.5
	8	Solving problems	2	25			3	16.7	5	18.5
(5) Challenges & exploration	9	Emergency & rescue					3	16.7	3	11.1
	10	Journeys					3	16.7	3	11.1
(6) People	11	About our lives	2	25			3	16.7	5	18.5
	12	Wishes & regrets	2	25	1	100	3	16.7	6	22.2
Total			8	100	1	100	18	100	27	100

Social/affective strategies are illustrated in Table 3, which shows minute emphasis in the textbook under study. Social/affective strategies are classified about 5 times in the group work strategy and only once in pair-work strategies.

**Table 3:** Frequencies and percentages of the infused social/affective strategies in the textbook.

Target English grade 9: student's book			Social/affective strategies					
			Pair work		Group work		Total	
Module	Unit		F.	%	F.	%	F.	%
(4) Critical thinking	7	Finding answers			2	40	2	33.3
	8	Solving problems						
(5) Challenges & exploration	9	Emergency & rescue			2	40	2	33.3
	10	Journeys						
(6) People	11	About our lives	1	100	1	20	2	33.3
	12	Wishes & regrets						
Total			1	100	5	100	6	100

### 4.2 Question 2: What is the approach of ninth grade teachers to adopt listening comprehension strategies?

Mean scores, standard deviation and degrees of listening strategies are revealed by Kuwaiti EFL teachers in Table 4. Higher extent of these categories was attributed from the analysis showing all the observed teaching units. However, the difference is elaborated from analysis in terms of rank order and mean scores. Cognitive strategy ought to be the most reliable strategy as it acquired highest mean score (M = 1.87, SD = 0.16); whereas, mean score and standard deviation of metacognitive strategies (M = 1.74, SD = 0.29) were lower than the cognitive strategies. The mean score and rank order of social/affective strategies were nearly equal to the metacognitive strategies (M = 1.75, SD = 0.26).

**Table 4:** Rank order, mean scores, standard deviations and degrees of listening strategies categories used by teachers.

No.	Rank	Dimension	Mean	Std. Deviation	Degrees
1	1	Cognitive strategies	1.87	0.16	High
2	3	Metacognitive strategies	1.74	0.29	High
3	2	Social affective strategies	1.75	0.26	High

The rank order and mean score for all the items have been revealed in Table 5. The results have indicated that majority of items comprised higher mean score while three items (12, 14 and 18) were moderate at mean score. “Focusing on listening for main ideas, then for details” were ranked first due to the highest mean score (M = 2.00, SD = 0.00). However, “helping pupils to work in groups for feedback” had the lowest mean score (M = 1.53, SD = 0.48).

**Table 5:** Rank order, mean scores, standard deviations and degrees of listening cognitive, metacognitive, and social/affective strategies used by the observed teachers.

Strategy	No.	Rank	Item	Mean	Std. deviation	Degrees
Cognitive strategies	1	8	Using linguistic clues to help pupils infer meaning, such as prefixes and suffixes.	1.79	0.23	High
	2	5	Paying attention to elaborate meanings in an English listening text to help pupils guess its meaning.	1.84	0.18	High
	3	6	Asking pupils to paying attention to summarizing key ideas that help in understanding the text better.	1.84	0.18	High
	4	3	Asking pupils to Write notes, messages, down on the board before giving an answer.	1.95	0.07	high
	5	1	Focusing on listening for main ideas, then for details.	2.00	0.00	High
	6	7	Highlighting the answers in the Pupils Book (e.g. the printed items, choices and pictures).	1.79	0.23	High
	7	4	Practicing and repeating the English words which students do not understand several times.	1.89	0.14	High
	8	2	Substituting words into other words to help pupils understanding.	1.95	0.07	High
	9	9	Using the words pupils understanding to guess the meaning of the words they don't understand.	1.79	.230	High
Metacognitive strategies	10	12	Helping pupils to have a plan in their mind before listening.	1.79	.230	High
	11	10	Helping pupils to pay attention to information they need.	1.89	.140	High
	12	14	Helping pupils to listen for key words.	1.63	.310	moderate
	13	13	Helping pupils to notice their mistakes.	1.68	.270	High
	14	15	Helping pupils to adjust interpretation if they realize that it is incorrect.	1.58	.410	Moderate
	15	11	Helping pupils to think back to everything heard to verify words and sentences meanings.	1.89	.140	High
Social affective strategies	16	16	Helping pupils to think back to see how they listened and about what I might be done differently next time.	1.89	.140	High
	17	17	Helping pupils to work in pairs for feedback	1.84	.180	High
	18	18	Helping pupils to work in groups for feedback.	1.53	.480	Moderate

### 4.3 Question 3: What is the congruence of listening strategies employed by English language teachers in the textbook?

The correspondence between the content “analysed” listening strategies and the “actual” teaching practices for EFL teachers has been demonstrated in Table 6. Spearman correlation uses ranks instead of assumptions about the distributions of the two variables. The results for spearman correlation in this study has revealed there is a moderate positive relationship between the variables (p = 0.667). However, there has been congruence between “analysed” and “observed” strategies rank scores characterized by the cognitive strategies. Therefore, it has been stated, that EFL textbook provides effective listening strategies in the ninth grade. Moreover, the actual practices of the teachers did not correspond towards listening comprehension strategies.

**Table 6:** Correlation Coefficient of the congruence between the ‘analysed’ and the ‘observed’ strategies.

Strategy	Rank of content	Rank of teacher observation	Spearman's Rho	Sig.
Cognitive strategies	1	1	0.50	0.667
Metacognitive strategies	2	3		
Social affective strategies	3	2		

## 5 Discussion

The listening comprehension strategies regarding learner's skills have been addressed through the stated research questions. The findings for the first research question have showed that cognitive strategies have had great prominence in the study; while, metacognitive strategies and social/affective strategies comparatively received lesser emphasis. Cognitive strategies were examined through 116 incidences in the textbook while 27 occurrences were revealed for metacognitive strategies. Furthermore, 6 occurrences were highlighted for social/cognitive strategies in the first research question. Therefore, it has been examined that textbook conveys listening strategies at a large extent. It has been revealed that Inferencing and practicing strategies were the most essential cognitive approaches occurring 5 times in each unit. Inferencing strategy has been deemed as one of the essential constituents throughout the listening activity to reveal the success degree of the ability. Reading, grammar patterns, imitation of sounds, and writing skills were prominent aspects revealed through the practice of sound strategy. Therefore, it has been appeared to have major impact on the learner's skills (Bidabadi and Yamat, 2013).

The most frequent metacognitive strategy examined from the content analysis has been the evaluation strategy, which illustrated 3 incidences in each teaching unit. Evaluation strategy is implemented into an entire language learning practice and has been realized to be dependent on the pedagogical sequence of planning, monitoring and evaluation. Therefore, listening learners are supported to maximally employ strategies for planning, monitoring, and evaluating their own understanding (Davoudi and Sadeghi, 2015). The most frequent social/affective strategy has been the group work strategy, which occurred once in each unit in all examined teaching units in the present study. The utilization of authentic dialogues has been illustrated from textbook analysis providing the mixture of all the approaches. It has been revealed that language skills are associated with the communicative interactive texts (Chien and Hsu, 2013).

The strategies involved in the development of influential listening awareness enhance the metacognitive comprehension of listeners. The significant strategies employed by students enable them to achieve particular assignments, expressing new strategies, and supplying more practical approach to the metacognitive strategies (Davoudi and Sadeghi, 2015). The elements of understanding and regulation of cognition are included in the aspects of metacognition. Similar to present study, another study conducted by Bidabadi and Yamat (2013) stated that declarative, conditional, and procedural knowledge is referred in the cognition knowledge; whereas, learners' thinking process is entailed in procedural knowledge. The evaluation of the incentive of learning strategies employ several different contents, which may include the selection of specific listening advances throughout the practice.

The present study has examined the extent of English language teachers using listening strategies in their actual and observed teaching practices, which resulted in high degree of utilization by teachers as 38 listening lessons were observed. Similarly, Ghanizadeh and Alishahi (2016) observed that the listening performance of Kuwaiti students escalated through listening strategies through organized listening lessons. Findings of the second research question in the present have been examined significantly through highest mean scores of cognitive strategies. The active control has been brought into concern through metacognitive strategies, which may surpass over the cognitive processes in the learning phase. Kuwaiti students were dominantly influenced through metacognitive strategies as it assists students to accomplish their objectives and decipher essence by evaluating learning comprehension. A study showed that social/affective strategies were also effective in the practice of teachers' skills and abilities. These attitudes are considered to be encouraging to perform better (Rahimirad & Zare-ee, 2015).

Liu and Thondhlana (2015) have found significant association among the listening comprehension strategies and listening skills of the students. The cognitive and metacognitive strategies are essential in determining the listening comprehension skills of students. Students have been observed to cover a satisfactory extent of metacognitive comprehension. The satisfactory extent has been provided by deeming great emphasis to the metacognitive strategy as it assists students to develop their learning skills as well as organize their learning activities. Therefore, listening comprehension strategies are realized to be providing a significant difference among more skilled and less skilled learners (Gilakjani, 2016). However, the present study has examined that these strategies allow listening learners to develop and attain better listening comprehension skills, better language efficiency, and an ability to eradicate task problems.

Problem solving skills have been recognized to be essentially resolved by the use of different comprehension strategies. Students are encouraged to implement their own experience and awareness to assume the essence of unknown phrases. Such planning and evaluation strategies are mostly associated with the satisfaction level of the students (Lee, 2016). It has been emphasized that students are more attentive towards their listening plans, establishing their own concepts, identifying their self-satisfaction, and evaluating their listening strategy significantly. The listening learners monitor Inferencing of texts and associate them with the text-emerging interpretation to acquire general emphasis (Janusik and Keaton, 2015).

In the present study, a lack of congruence has been determined from the content analysis between the observed and analysed metacognitive strategies. It has been assessed that the difference among spoken and written language might be associated to the paucity of congruence between the strategies, which has been analysed and observed for the study. However, another study examined that extensive use of directed attention strategies tend to be more associated with the student's satisfaction (Yeldham and Gruba, 2016). Therefore, listening learners are identified to store a potential to endure their focus whenever they get distracted.

Complex structure and vocabulary items have been found to be firmly associated with the written texts. Therefore, the findings of the present study illustrated no difference between the spoken and written texts. These students have also been observed to be more attentive to manage their complexities in comprehending texts instead of quitting the task (Dahlberg, 2016). This difficulty is presumed to emerge in the state of anxiety and self-confidence towards the listening practice of the language. The lack of correspondence among the observed and analysed strategies has implicated to stress upon strategy based instruction, which has been identified as a factor that may enables them to apply the listening strategies effectively (Vandergrift and Baker, 2015).

## 6 Conclusions

The study findings have concluded that explicit strategy training should be incorporated in national listening curriculum in Kuwaiti schools to endow teachers with some guidelines to enhance learners' listening comprehension. Language listening comprehension strategies have been determined as an essential requirement for Kuwaiti ninth grade students. The results were effective in illustrating that instruction materials and systematic attention are required for strategy development. The examination of the textbooks revealed that categories of listening strategies are focused in all units of ninth grade. Cognitive strategies, metacognitive, and social/affective strategies are essential to establish appropriate practices from the textbook. Better opportunities are endowed by content analysis for the EFL teachers to assist students employing cognitive, metacognitive and social/affective strategies significantly. On the contrary, significant opportunities failed to be established from textbook listening strategies for EFL teachers. Furthermore, the findings did not reveal any statistically significant differences among analysed and practiced strategies.

Listening comprehension has been regarded as an active and sentient process, which is viable for listeners to receive and process the input, store the information, and decode it. These skills are enhanced through significant role of metacognition. Planning, monitoring, evaluating and problem-solving strategies are the most dominant metacognitive strategies. The study findings are appropriate for endowing teachers with some guidelines to enhance learners' listening comprehension. It has been recommended that explicit strategy training should be incorporated in national listening curriculum in Kuwaiti schools. Teacher-training program need to be implemented in Kuwaiti schools.

The concept of implementing these training programs can provide awareness regarding effects of listening comprehension strategies on learner's progress. Further, more concentration must be provided to infuse interesting listening activities. It has been suggested to devise a language textbook that entails assorted constituents of listening strategies. The needs, principles, content, goals, setting and assessment should be considered when devising the textbook. Cognitive, metacognitive and social/affective strategies must be infused among students through innovative ways. Teaching process entails supportive classroom dynamics and language use; therefore, it is considered as a multi-dimensional strategy. The development of teachers' teaching practices is attained through strategy-based textbooks. However, future studies need to include quantitative approach with larger sample size to examine the impact of listening comprehension strategies on enhancing the learner's skills. Quantitative findings will be appropriate in determining the impact of these strategies on learner's abilities. Furthermore, visual tools must be adopted by teachers to develop listening skills of students and to entail them in interactive listening conversations.

## Declarations

### Author contribution statement

Asma F.T Al-Azzemy, Dina A.H Al-Jamal: Conceived and designed the experiments; Performed the experiments; Analyzed and interpreted the data; Contributed reagents, materials, analysis tools or data; Wrote the paper.

### Funding statement

This research did not receive any specific grant from funding agencies in the public, commercial, or not-for-profit sectors.

### Competing interest statement

The authors declare no conflict of interest.

### Additional information

No additional information is available for this paper.
